# Intrinsic and synaptic regulation of axonal excitability in dopaminergic neurons

**DOI:** 10.3389/fncel.2025.1681044

**Published:** 2025-11-06

**Authors:** Jackie Seddon, Paul F. Kramer

**Affiliations:** 1 Neuroscience Graduate Program, University of Michigan, Ann Arbor, MI, United States; 2 Department of Molecular, Cellular, and Developmental Biology, University of Michigan, Ann Arbor, MI, United States

**Keywords:** dopamine, axons, neurophysiology, ion channels, modulation, excitability, receptor signaling

## Abstract

Dopamine released from the axon terminals of dopaminergic neurons is central to behaviors like reward learning and complex motor output. The dynamic control of dopamine release canonically occurs through two main mechanisms: the modulation of somatic excitability and the regulation of vesicular release at presynaptic boutons. However, there is also a third mechanism: the precise and local control of axonal excitability. Together, these three mechanisms control the amplitude and timing of dopamine release from terminal axons. In this review, we examine the intrinsic properties and dynamic modulation of dopaminergic axons. First, we will examine their intrinsic properties, including membrane biophysics and morphological features. Second, we will focus on the modulation of axonal excitability through receptor signaling. Finally, we will review how drugs of abuse directly influence axonal physiology, and how axonal excitability influences the progression and etiology of Parkinson’s disease. Through this review we hope to highlight the important role that modulation of axonal excitability plays in controlling dopamine release, beyond action potential propagation.

## Introduction

Axons are often conceptualized as a relatively simple component of the neuron. According to the canonical view, the purpose of an axon is to propagate action potentials (Hodgkin and Huxley, 1952). However, this understanding began to evolve through studies on circuits in *Cancer borealis* when researchers found that focal applications of transmitter to peripheral axons could evoke action potentials along almost the entire axon (Goaillard et al., 2004; Bucher et al., 2003; Meyrand et al., 1992). In the past three decades, more dynamic processes have been discovered in axons including inhibition, gating of branch point propagation, analog modulation, and synaptic-like excitation. Here we will focus on dopaminergic neurons of the basal ganglia, where axonal signaling and local modulation have been found to be critical controllers of neurotransmitter release and network activity (Kramer et al., 2022; Rice and Cragg, 2004).

The basal ganglia is a set of interconnected subcortical nuclei regulating both voluntary movements and reward learning. Broadly, the nuclei of the basal ganglia process descending cortical information to generate desired locomotor behavior. The two principal input regions of the rodent basal ganglia are the nucleus accumbens (NAc) and the dorsal striatum (DS), which together make up the striatal complex. These regions also receive dense inputs from midbrain dopaminergic neurons, which regulate basal ganglia activity through the release of dopamine. The NAc receives dopaminergic input from the ventral tegmental area (VTA) via the mesolimbic pathway. This pathway is implicated in the processing of rewards and associative learning (Yates, 2023). The DS, which consists of the caudate and putamen nuclei in primates, receives input from the substantia nigra *pars compacta* (SNc) via the nigrostriatal pathway. This pathway is implicated in motor actions.

Dopaminergic axons make incredibly dense connections with local circuits and interneurons in the DS and NAc. The axon of a single SNc dopaminergic neuron can create between 102,165 and 245,103 synapses in the rat DS (Matsuda et al., 2009). Though, recent evidence indicates that only about 1/3 of those varicosities may actively release dopamine (Liu et al., 2018). For proper circuit function, dopamine release needs to be tightly controlled in timing, amplitude, and space. Some of this control is exerted by modulation of action potential output from the cell bodies. But there is also another layer of control exerted by direct modulation of dopamine release from the axon. It is well understood that inputs onto the axon can regulate the release of dopamine through modulation of vesicular release machinery (Sulzer et al., 2016). However, less appreciated is the notion that inputs can also modulate local axonal excitability to control the action potential waveform and its propagation.

The intrinsic properties of axons are conducive to the transmission of action potentials. They express high densities of voltage-gated channels and have a high input resistance, often exaggerated by the presence of myelin. Optimizing for action potential propagation produces local biophysical properties that make axons unique from the soma and dendrites, with distinct mechanisms regulating excitability. For example, because axons have a high input resistance, even small currents across the plasma membrane can cause large fluctuations in the membrane potential. These currents may arise from voltage-gated channels, electrogenic transporters, or ligand-gated ion channels. Notably, neurotransmitter-activated currents on axons permit dynamic, localized modulation of excitability. Many types of modulators can affect dopaminergic signaling, including endogenous hormones and environmental substances. As a result, the dopaminergic system faces the challenge of maintaining normal function and supporting appropriate reward processing, motor output, and motivation, despite constant physiological and environmental fluctuations. As our understanding of how endogenous and exogenous modulators affect dopaminergic neurons expands, it is important to consider that these substances may act differently on axons than on somas and dendrites.

This review will summarize the current state of what is known about modulation and control of dopaminergic axonal excitability (Table 1). We will first examine the intrinsic excitability of dopaminergic axons, followed by the inputs that modulate this initial state. Through this discussion, we hope to draw attention to the mechanisms by which action potentials in the axon may be modulated or locally initiated following ligand-gated receptor activation. We will end by addressing the potential relevance of these findings as they relate to diseases and disorders associated with dopaminergic axons.

**Table 1:** The key channels and receptors that are known to control dopamine release through modulation of axonal excitability.

Channel types		Effect on dopamine transmission	Mechanisms in the axon	Region	References
K^+^	K_v_1.2	Activation inhibits release	Action potential kinetics and repolarization via D-type currents	DS	Xiao et al. (2021)
	K_v_1.4	Activation inhibits release	Action potential kinetics and repolarization via A-type currents	DS	Xiao et al. (2021)
	SK	Activation inhibits release	Unknown	DS	Zhang and Sulzer (2003)
	K-ATP	Activation inhibits release	Unknown	DS	Patel et al. (2011)
Na^+^	Na_v_	Activation promotes release	Resting membrane potential controls Na_v_ availability through inactivation.	DS, NAc	Kramer et al. (2020) and Xiao et al. (2021)
	Na_v_1.2	Knockout reduces dopamine release	Reduced action potential height and Na^+^ currents	VTA, NAc	Li et al., 2025
Ca^2+^	L-type	Activation promotes release	Unknown	DS	Brimblecombe et al. (2015)
	T-type	Activation promotes release	Unknown	DS	Brimblecombe et al. (2015)
	N-type	Activation promotes release	Action potential-depedent Ca^2+^ entry	DS, NAc	Brimblecombe et al. (2015) and Sgobio et al. (2014)
	P/Q-type	Activation promotes release	Action potential-depedent Ca^2+^ entry	DS, NAc	Brimblecombe et al. (2015) and Sgobio et al. (2014)

Neuromodulator	Receptors	Effect on dopamine release	Mechanism (hypothesized or known)	Region	References
Acetylcholine	nAChR	Activation can promote or inhibit release	Excitation of distal axons and action potential initiation. Unknown mechanism of inhibition.	DS, NAc	Kramer et al. (2022), Liu et al. (2022), and Zhang et al. (2025)
	M1-like mAChR	Activation potentiates release	Unknown	NAc	Shin et al. (2015)
	M2- like mAChR	Limits dopamine release in a frequency dependent manner	Decreased ACh release and reduced nAChR activation on dopaminergic axons.	NAc shell	Shin et al. (2017)
GABA	GABA_A_	Activation inhibits release	Na + channel inactivation from depolarization and shunting inhibition	Medial forebrain bundle, DS	Kramer et al. (2020)
	GABA_B_	Activation inhibits release	Unknown	DMS	Holly et al. (2021)
Dopamine	D2R	Activation inhibits release	Potentiation of K_v_1 currents and inhibition of Ca2 + currents	DS, NAc	Martel et al. (2011) and Sgobio et al. (2014)
	DAT	Activation reduces short term depression	Electrogenic current alters resting potential	DS, NAc	Condon et al. (2019)

If known, the underlying mechanism is also summarized.

## Intrinsic control of excitability in dopaminergic axons

**Figure 1:**
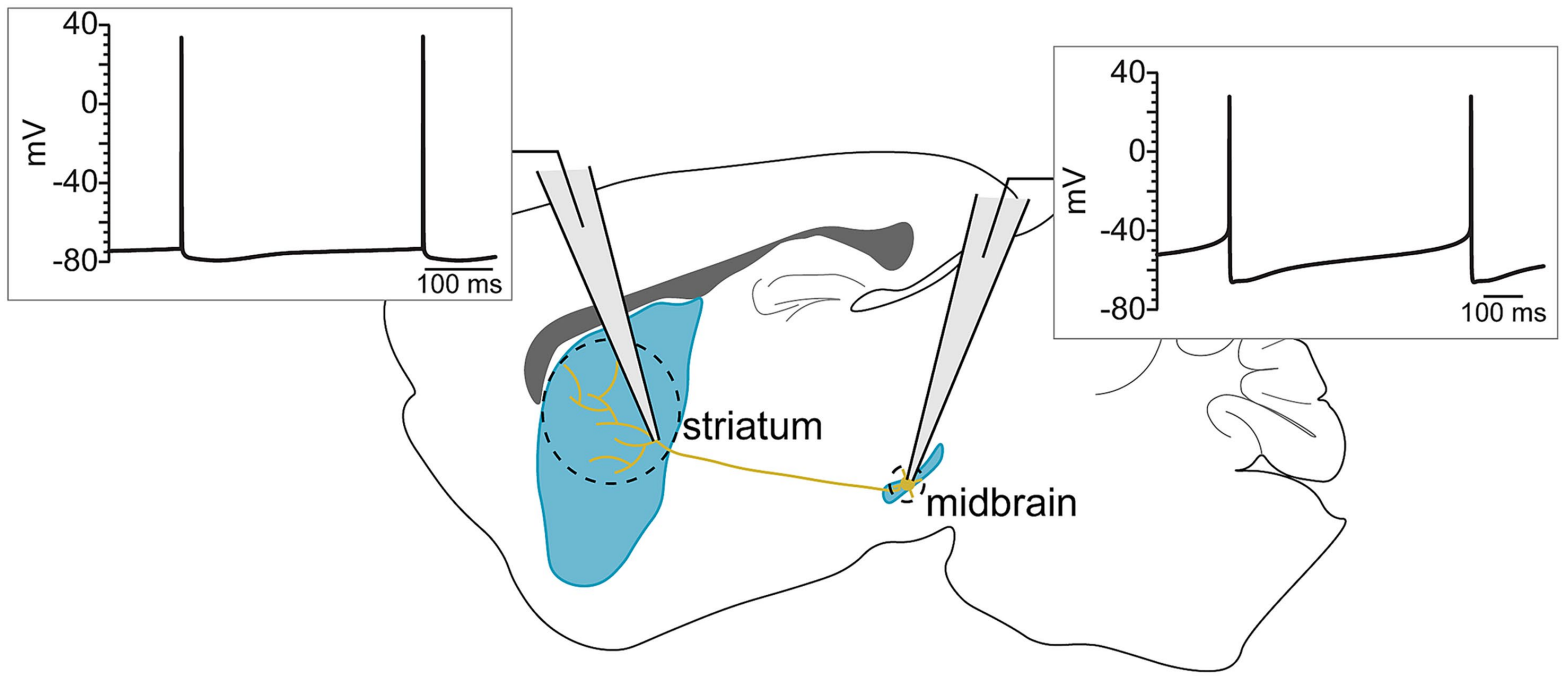
Somatic and axonal action potentials have distinct waveforms, reflecting the different ion channel currents that mediate their conduction across the length of the axon.

The functional result of ligand-gated receptor activation or inhibition in dopaminergic axons will depend on the axon’s intrinsic properties. Therefore, before examining the modulation of dopaminergic axons, it is necessary to first summarize what is known about the intrinsic excitability of this compartment. Axonal excitability is set by the expression of ion channels, which also determine the shape of the action potential waveform. Dopaminergic neurons are characterized by a well-known action potential waveform that is wide, with a large after-hyperpolarization and a substantial interevent slope (Puopolo et al., 2007; Grace and Bunney, 1984). However, these features recorded from the soma are absent or reduced in dopaminergic axons (Kramer et al., 2020). This observation highlights the fact that the axons of dopaminergic neurons express a different complement of ion channels from the soma and dendrites, thus producing a distinct action potential waveform (Figure 1). In this portion of the review, we will discuss what is known about the intrinsic excitability of dopaminergic axons.

### Ion channels

#### K^+^ channels

Potassium channels provide the principal repolarizing drive of action potentials and govern excitability across subcellular domains by virtue of their heterogeneous kinetics and distribution. They are gated by diverse triggers—voltage changes, intracellular Ca^2+^ and Na^+^, G-protein activation, ATP availability, and other intracellular signals—such that each K^+^ channel variant distinctly influences neuronal excitability and the waveform of the axonal action potential.

One study examining the role of voltage-activated K^+^ (K_v_) channels in modulating dopamine release found that K_v_1.2 co-immunoprecipitated with dopamine D2 receptors (D2Rs) in samples from mouse striatal tissue, demonstrating a physical interaction between these proteins. Dopamine D2Rs are inhibitory G protein-coupled receptors that, when activated, reduce vesicular dopamine release. Functionally, it was found that D2R-mediated inhibition was attenuated by blockers of K_v_1.1, −1.2, and −1.6 (Fulton et al., 2011). Together, these data were interpreted to mean that autoregulatory inhibition by D2 receptors is partially mediated by the activation of K_v_1 channels. During action potential firing in dopaminergic axons, both K_v_1.2 and K_v_1.4 channels repolarize the membrane and are therefore critical to determining impulse duration. Interestingly, the axonal resting membrane potential regulates the availability of these channels, meaning that a subthreshold depolarization leads to their inactivation, and thus a wider action potential waveform (Xiao et al., 2021).

SK channels are calcium-activated K^+^ channels, primarily studied in the soma of dopaminergic neurons where they regulate intrinsic firing patterns, the development of their characteristic intrinsic electrical phenotype (Dufour et al., 2014a; Dufour et al., 2014b), and can be activated by glutamate to inhibit activity (Morikawa et al., 2000; Fiorillo and Williams, 1998; Deignan et al., 2012; Wolfart et al., 2001). The mechanism of this inhibition by glutamate is the activation of metabotropic glutamate receptors (mGluRs) that triggers Ca^2+^ release from stores, which in turn activates SK. In the axons of DS dopaminergic neurons glutamate has similar inhibitory effects. Specifically, spillover from cortical terminals activates mGluR1 on dopaminergic neuron axons to suppress dopamine release through SK activation (Zhang and Sulzer, 2003). The mechanism for this reduction in dopamine release is unknown but may have to do with a hyperpolarization of the axonal membrane potential, similar to the somatic mechanism.

Dopaminergic axons are energetically costly due to their large size and tonic spiking activity (Bolam and Pissadaki, 2012). This is relevant to the progression of Parkinson’s disease, where a central phenotype is mitochondrial dysfunction (Swerdlow et al., 1996; Mattson et al., 2008; Biskup and Moore, 2006). It is therefore important to understand how axonal ATP affects cellular activity. In addition to providing a source of energy, ATP is also an important factor in modulating excitability in dopaminergic axons. In the DS, the physiological stimulation of H_2_O_2_ signaling in brain slices inhibits dopamine release by binding to ATP-sensitive K^+^ (K-ATP) channels (Avshalumov et al., 2003; Avshalumov and Rice, 2003) present on the dopaminergic axons (Patel et al., 2011). Further elucidation of this mechanism—particularly ATP/H₂O₂ interactions with neuromodulators—is essential for understanding the interplay between axonal degradation, excitability, and cellular energy management.

Large-conductance calcium-activated K^+^ (BK) channels are also central regulators of neuronal excitability. The location of BK channels has been shown to have a critical impact on neuronal firing. BK channels are localized to axons and nerve terminals (Misonou et al., 2006), as well as to the soma and dendrites. In the dopaminergic neuron somas of the SNc, BK channels are major contributors to spike repolarization, and inhibition of BK leads to a widening of the action potential (Kimm et al., 2015). However, their role in determining the kinetics of the axonal action potential is not known, though BK channels may play a role in axonal degradation associated with neurodegenerative diseases and autophagy (Kochlamazashvili et al., 2025).

#### Na^+^ channels

Voltage-gated sodium (Na_v_) channels mediate the rapid upstroke of action potentials and their propagation. Action potentials are initiated at the axon initial segment (AIS): a specialized, high-density Na_v_ domain that defines the onset of the axonal compartment. In myelinated axons, action potential conduction is supported by Nodes of Ranvier (Jenkins and Bender, 2025). However, the long dopaminergic axons that extend from the SNc and VTA are unmyelinated or poorly myelinated (Nirenberg et al., 1996). Therefore, the mechanisms of action potential conduction in these axons exhibit unique features that distinguish them from myelinated axons. In this section, we will discuss potential intrinsic mechanisms by which Na^+^ channels contribute to axonal excitability and action potential propagation.

In dopaminergic axons, it is unclear if Na_v_ channels cluster, or if they are distributed without a regulated pattern. Some unmyelinated axons have been shown to have clustered Na_v_ channels, such as in *Aplysia* (Johnston et al., 1996). Modeling studies have explored the propagation characteristics of unmyelinated axons with various conductance distributions. Including a slow-inactivating gate on axonal Na_v_ channels improved the faithful propagation of action potentials. Notably, this effect was frequency dependent, resulting in a tradeoff between high-frequency firing and reliable propagation (Zang et al., 2023). These findings implicate slow-inactivating Na^+^ currents in the regulation of excitability in unmyelinated axons.

There are biophysical differences between axonal and somatic Na_v_ channel kinetics in dopaminergic neurons. In both the SNc and the VTA, axonal Na_v_ channels take longer to inactivate during small depolarizations at subthreshold potentials. They are also faster to recover from this inactivation than their somatic counterparts (Yang et al., 2019). However, there remain unanswered questions about Na_v_ channels in more distal axon regions of the striatal complex. Recent work highlights the importance of Na_v_1.2 in both the main axon trunk of VTA dopaminergic neurons and in distal axon terminals in the NAc. Na_v_1.2 loss decreased dopamine release from NAc axon terminals elicited by either local carbachol application or electrical stimulation (Li et al., 2025).

The availability of axonal Na_v_ channels can be rapidly modulated because of their fast inactivation kinetics. This makes many features of the axonal action potential dependent on subthreshold membrane potential oscillations. In a hippocampal model, fast pulses of hyperpolarization disinhibit Na_v_ channels, leading to increased spike amplitude in the axon (but not in the soma) (Rama et al., 2015). This suggests that hyperpolarizing inputs from local interneurons to the axon may, in effect, be excitatory through this disinhibition. These findings highlight the digital-analog action potential theory, a central framework for understanding axonal excitability (Zbili and Debanne, 2019).

#### Ca^2+^ channels

Dopaminergic neurons express L-type, T-type, N-type, and P/Q-type voltage-gated calcium channels (VGCCs). In the soma of dopaminergic neurons, low-voltage-activated T-type and L-type channels contribute to pacemaking, whereas high-voltage-activated L-type, N-type, and especially P/Q-type channels mediate action potential-evoked Ca^2+^ influx and dopamine release (Mintz et al., 1992; Sutton et al., 1999). Removing both P/Q-type and N-type Ca^2+^ channels in dopaminergic neurons reduces action potential-evoked neurotransmitter release (Liu et al., 2022).

Interestingly, VGCC function has been reported to differ between the DS and NAc. Dopamine release in the DS was reduced following individual inhibition of P/Q-type, N-type, T-type, and L-type VGCCs (Brimblecombe et al., 2015). However, the role of T-type and L-type VGCCs in mediating DS dopamine release is unsettled. A separate study found that presynaptic Ca^2+^ entry in DS dopaminergic axons is dependent on N- and P/Q- type VGCCs, while L-type channels did not significantly contribute (Sgobio et al., 2014). In contrast to the DS, inhibiting L-type and T-type VGCCs in the NAc had little effect on dopamine release (Brimblecombe et al., 2015). Additionally, there may be differences in Ca^2+^-dependent dopamine release between the axonal terminals and the somatodendritic compartment. For example, the somatodendritic release of dopamine is only partially dependent on VGCC-mediated Ca^2+^ entry (Chen et al., 2006).

Additionally, axonal D2Rs and acetylcholine receptors (AChRs) directly influence presynaptic Ca^2+^. The muscarinic AChR (mAChR) agonist oxotremorine reduced the amplitudes of Ca^2+^ transients by 75%, and the dopamine D2R agonist quinpirole caused a concentration-dependent inhibition of evoked Ca^2+^ transients (Sgobio et al., 2014). These data demonstrate dynamic actions of mAChRs and D2Rs in controlling Ca^2+^ in the presynaptic axonal terminals.

### Morphology

#### Axonal branching

The architecture of striatal dopaminergic axons is unique in its highly branched structure, with a field of overlapping axons from other dopaminergic neurons and from local striatal neurons. In the mouse, dopaminergic axons were measured to have an average total length of 467,000 μm, with bifurcations of the axon occurring about every 31 μm (Matsuda et al., 2009). This produces an estimated total of 15,000 branches per dopaminergic neuron in mice, which is substantial, though likely an underestimation what occurs in human neurons (Kramer et al., 2020). This presents an interesting problem for the propagation of action potentials at branch points in these highly variegated structures. One study suggests that the high degree of branching in dopaminergic axons leads to attenuation of action potentials as they propagate, due to GABA_A_ receptor activation. This suggests that, as action potentials travel through dopaminergic axons, they are subject to inhibition via tonic GABA_A_ activity, leading to shortened action potentials and reduced dopamine release (Kramer et al., 2020). The shortened amplitude of the action potential may also lead to increased branch point failures, though further investigation into this issue remains necessary. Interestingly, GABA_A_ activity in axons is not always inhibitory. In the spinal cord, GABA_A_ activity specifically near nodes and branch points facilitates propagation through transient depolarizations, providing regulation of action potential conduction and enhancing the computational power of this system (Sgobio et al., 2014). Furthermore, in the cerebellum, axonal GABA_A_ receptors have been found to potentiate glutamate release from granule cells onto Purkinje neurons (Sgobio et al., 2014).

These contrasting results of GABA_A_-dependent modulation of axonal excitability are intriguing and remain unexplained. Possible mechanisms mediating these differences could stem from axonal morphology. Dopaminergic axons are highly variegated whereas cerebellar granule cells and sensory axons in the spinal cord are less branched. Or it could stem from the kinetics of GABA_A_ receptor activation. In dopaminergic axons GABA_A_ is predominately a tonic signal, without a stimulated component yet described (Kramer et al., 2020), whereas in the spinal cord the receptors are transiently activated by interneurons.

#### Myelination

Dopaminergic axons of the ventral midbrain are often described as being unmyelinated, poorly myelinated, or lightly myelinated. Early work in the 1960s described dopaminergic axons extending from the SNc to the striatum as thin (smaller than 0.3 μm in diameter) and “poorly myelinated” due to a slow action potential conduction velocity recorded *in vivo* (York, 1970). Furthermore, early electron micrographs of dopaminergic axons displayed a lack of the membrane ensheathments that are characteristic of myelin (Nirenberg et al., 1996). However, a recent study finds that roughly 86% of VTA dopaminergic neuron axons are myelinated. Moreover, the authors suggest that the myelination of dopaminergic axons is a dynamic process, responding to increases in activity and exposure to opioids. Interestingly, changes in myelination state were not observed in medial forebrain bundle or in the NAc (Yalçın et al., 2024). It is therefore unsettled whether these axons are all partly myelinated, if there are subpopulation differences in their myelination states, regional differences in myelination, or some combination of these possibilities. What is clear is that the axons of dopaminergic neurons are not all heavily myelinated.

#### Axon initial segment

The canonical understanding of action potential propagation often fails to capture complicated dynamics between different compartments of the neuron. The AIS is a critical region of the axon where action potentials initiate (Debanne et al., 2011). This unmyelinated portion of the neuron is usually located proximal to the soma, and expresses the highest density of Na_v_ channels (Bean, 2007). Interestingly, dopaminergic neurons have an AIS that extends from a thick basal dendrite (Häusser et al., 1995). Importantly, differences in ion channel kinetics at the AIS differ across brain regions, with important ramifications on action potential shape and propagation (Bean, 2007).

The AIS may also influence the spontaneous activity of dopaminergic neurons, though this hypothesis requires further examination. In one study, researchers found that dopaminergic neurons with faster firing rates tended to have larger and more proximal AIS regions. Computational modeling further suggests that the AIS and soma may function as independent, spatially separated Na_v_1 oscillators that synchronize their activity at a common frequency (Meza et al., 2018). However, other research has shown that spiking frequency and action potential shape in dopaminergic neurons is independent of AIS length or distance from the soma. Rather, the excitability and morphology of the axon-bearing dendrite is what was found to define the frequency and kinetics of dopaminergic action potentials (Moubarak et al., 2022; Moubarak et al., 2019). This coupled oscillator model suggests that the axon is not involved in the pacemaking of dopaminergic neurons (Wilson and Callaway, 2000; Jang et al., 2014). While the role of the AIS in regulating pacemaker activity is debated, the distal axon is unlikely to be a part of the coupled oscillator. Voltage recordings from distal, soma-isolated, dopaminergic axons display no intrinsic oscillatory activity (Kramer et al., 2022; Kramer et al., 2020).

#### Organization of axonal ion channels

Dopaminergic axons are mostly unmyelinated or lightly myelinated. Therefore, many of these axons lack discrete nodal regions that typically organize ion channels. In fact, the organization and biophysical properties of distal dopaminergic axon ion channels are mostly uncharacterized. Systematic mapping of channel distribution and clustering is therefore essential for revealing how these axons maintain high-speed conduction and control of local excitability. This is especially relevant during high-frequency bursting where branch point failures are more common (Ofer et al., 2024), and which have recently been shown to occur in the axons of dopaminergic neurons as well (Yee et al., 2025).

Unmyelinated axon fibers still express organizational proteins that form structures, even in the absence of nodes. One study observed that actin, one of the main structural proteins in the axon, forms ring-like formations around the circumference of the axon. These rings were found to be evenly spaced along the length of the axon, from about 180 to 190 nm apart, and were not observed in dendrites (Xu et al., 2013). Atomic force microscopy of the axonal plasma membrane found that axons are about 6-fold stiffer than the soma, and 2-fold stiffer than dendrites (Zhang et al., 2017). These stiff actin structures have been hypothesized to serve as a scaffold for organizing ion channels along the axon.

Another possible model of ion channel organization in the axon is one of clustered Na_v_ channels on lipid rafts. Lipid rafts are regions of protein and lipid assemblies, enriched with sterol sphingolipids (Pike, 2006), which can contribute to functional localization of proteins on the plasma membrane. Additionally, they have been found have an impact on cell excitability (Pristerà et al., 2012). The model indicates that lipid raft confinement of Na_v_ channels mediates micro-saltatory conduction of action potentials, mirroring the function of Nodes of Ranvier. Additionally, this model suggests that the lipid rafts do not pose any significant increase in metabolic cost or propagation velocity (Neishabouri and Faisal, 2014).

#### Terminal release structures

Dopaminergic terminals express some of the same synaptic scaffold proteins as excitatory synapses that are essential for action potential triggered, and Ca^2+^-dependent, neurotransmitter release (Liu et al., 2018; Banerjee et al., 2020). These include RIM, RIM-BP, Munc13, Bassoon/Piccolo, ELKS, and Liprin-*α*. Of these proteins, Munc13 and RIM closely interact with dopamine-filled vesicles, priming them for fusion. However, in other ways, dopaminergic release sites differ from classical glutamate synapses. ELKS and RIM-BP are not required, and RIM, Munc13, and Liprin-α support scaffold structures (Banerjee et al., 2022). Once dopamine is released from the terminal, it signals with high temporal precision for metabotropic signaling (Howe and Dombeck, 2016). Dopamine receptor activation occurs rapidly in response to a high concentration of neurotransmitter localized to discrete areas (Gantz et al., 2018; Courtney and Ford, 2014). These features endow striatal dopamine transmission with focal characteristics that cannot be fully explained by volume-transmitted release.

## Receptor-mediated modulation of axonal excitability

**Figure 2:**
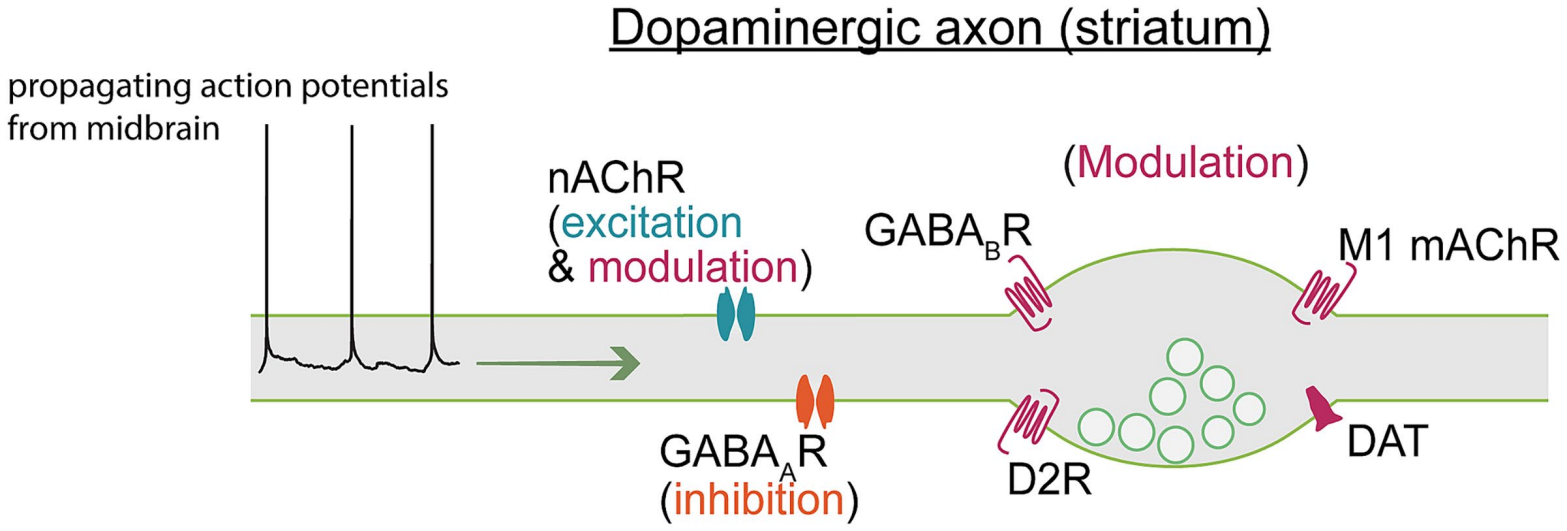
Axon terminals express ligand-gated receptors and electrogenic transporters that modulate axonal excitability. Action potentials propagating from the soma therefore traverse a landscape of shifting excitability states that may produce regionally distinct dopamine release, even within a single axon. A full breakdown of channels and receptors modulating axonal excitability can be found in Table 1.

Modulation of neuronal circuits via hormones, peptides, and neurotransmitters enables fine-tuned signal adaptations. Many studies examining the effects of endogenous modulators and exogenous drugs on neuronal excitability have been focused on responses in the soma due to its experimental accessibility. Some studies have been able to access the AIS, but few have been able to record the properties of distal axons. Of particular interest is the role of neurotransmitters that activate ion permeable receptors along the axon. In this section, we will discuss what is known about how neurotransmitters, and exogenous drugs, alter the excitability of dopaminergic axons to locally control dopamine release (Figure 2).

### Endogenous modulators

#### Cholinergic signaling

There has been growing interest in the relationship between dopamine and acetylcholine in the basal ganglia. Acetylcholine released by cholinergic interneurons (CINs) onto dopaminergic axons can both modulate and evoke dopamine release (Adrover et al., 2020; Mohebi et al., 2019; Cachope and Cheer, 2014). However, incongruities in the literature remain about the precise influence of CIN-mediated control of dopamine release in the DS and NAc regions. Nicotinic acetylcholine receptors (nAChRs) serve as crucial molecular mediators coupling cholinergic signaling to the regulation of dopamine release. These are ionotropic receptors that cause transient depolarizations in dopaminergic axons. In *ex vivo* brain slices, it has been shown that activation of axonal nAChRs can trigger action potentials in the distal axonal compartment to evoke dopamine release (Kramer et al., 2022; Liu et al., 2022; Threlfell et al., 2012). Importantly, individual CINs are able to trigger local release of dopamine *ex vivo* (Matityahu et al., 2023), suggesting a precise control of dopamine by striatal CINs. These findings strongly support the conclusion that CINs excite dopaminergic neurons, and evoke dopamine release, through an axo-axonic connection that relies on nAChR signaling. However, the role of nAChR signaling in modulating dopamine release is not settled. One recent study suggests that small, putative subthreshold nAChR-mediated depolarizations in the dopaminergic axons may inhibit dopamine release (Zhang et al., 2025). Additionally, CIN pauses are required to induce long-term potentiation, suggesting a decrease in acetylcholine release facilitates dopaminergic signaling (Reynolds et al., 2022).

These incongruities seen in *ex vivo* brain slices are reflected also by experiments performed *in vivo*. In behaving animals, dopamine moves across the DS in a wave-like fashion, with opposing dopamine waves exerting some control over behavioral reward prediction (Hamid et al., 2021). Acetylcholine is mainly released from a sparse population of CINs, and surprisingly also moves across the DS in a similar wave-like fashion to dopamine. What’s more, activity in the cholinergic neuropil of the DS is highly synchronized (Matityahu et al., 2023). Combined, these studies support the conclusion that one key function of CINs is to directly evoke dopamine release. However, other research has found no causal link between CINs and dopamine release in the DS. In the dorsolateral striatum, blocking nAChR activity, or knocking out the β2 nAChR subunit, did not meaningfully alter dopamine activity (Krok et al., 2023). Furthermore, eliminating acetylcholine release in the ventrolateral striatum did not alter reward-related dopamine signals, while elimination throughout the whole striatum had a profound effect (Chantranupong et al., 2023). Finally, in defined dopaminergic subpopulations across the whole striatal complex, somatic firing closely correlates with axonal Ca^2+^ transients, suggesting minimal local control of release (Azcorra et al., 2023).

Together, these findings reveal a complication to the causal relationship between acetylcholine signaling and dopamine release. The mechanistic data from *ex vivo* brain slices show that cholinergic inputs to dopaminergic axons are well established to dynamically modulate—and evoke—dopamine release (Rice and Cragg, 2004). However, how and when this occurs *in vivo* to influence specific behaviors remains unclear. Given the phasic, coordinated release of dopamine and acetylcholine in the striatal complex, elucidating how these signals interact with voltage-gated ion channels in the axon is therefore essential to understanding the relationship between these two critical modulators of basal ganglia activity.

In addition to nAChRs, muscarinic acetylcholine receptors (mAChRs) are abundantly expressed throughout the striatum and exert receptor subtype-specific modulation of dopamine release that varies with firing frequency and across striatal subregions (Threlfell et al., 2010; Shin et al., 2015). CINs express mAChR autoreceptors on their terminals that reduce acetylcholine release, resulting in modulation of nAChR activity (Threlfell et al., 2010). In one study, it was found that mAChR signaling limited dopamine release in a frequency-dependent manner that also depended on the activity of nAChRs on dopaminergic axons in the NAc core but not the NAc shell (Shin et al., 2017). Dopaminergic axons also express excitatory mAChRs that enhance dopamine release, though the mechanism remains unclear (Threlfell et al., 2010; Shin et al., 2015). In one study, the M1 subtype of the mAChR was found, in oocytes, to inhibit GIRK1 and GIRK4 channels, thus increasing excitability (Hill and Peralta, 2001). GIRK channels are abundantly expressed in dopaminergic axons (Hobson et al., 2022), leading to the hypothesis that M1 mAChRs may act through similar mechanisms to enhance dopamine release.

#### GABAergic input

GABA is co-released from some dopaminergic terminals (Tritsch et al., 2016; Borisovska et al., 2013) and GABA receptors are expressed along the axon and its terminals (Kramer et al., 2020; Tritsch et al., 2016; Kim et al., 2023). Functionally, GABA release may serve as an autoinhibitory signal to inhibit dopamine release following trains of action potentials (Kim et al., 2023). Also, GABA released from striatal neurons may act as an inhibitory signal that reflects network activity. The mechanisms of GABA_A_ inhibition in axons have long been debated. Axonal GABA_A_ receptors in dopaminergic neurons are depolarizing, a finding that seems counter to their inhibitory function. GABA_A_-mediated inhibition of dopamine release involves two mechanisms: Na_v_ channel inactivation secondary to depolarization (Rama et al., 2015), and shunting inhibition (Kramer et al., 2020; Xia et al., 2014). This dual mechanism of inhibition may be cell type specific, as GABAergic axo-axonal signals in cerebellar parallel fibers are depolarizing and increase neurotransmitter release (Pugh and Jahr, 2011).

GABA may also act to regulate the excitation of dopaminergic neurons by shunting nAChR inputs (Brill-Weil et al., 2024) to reduce stimulated dopamine release (Kramer et al., 2020). The dense GABAergic architecture of the striatal complex poses significant challenges to accurately localizing and characterizing inhibitory pathways. It has been observed that striatal CINs do not receive GABA from SNc axons (Straub et al., 2014), but rather from local GABAergic interneurons (Dorst et al., 2020), afferent inputs (Brown et al., 2012), and perhaps even from other CINs (Lozovaya et al., 2018). However, in this study the authors show that antagonizing GABA_A_ receptors enhances nAChR-mediated excitation of dopaminergic axons without altering local striatal acetylcholine levels. These findings suggest that the effects of GABA on dopaminergic axonal excitability are not mediated by broader circuit mechanisms. Instead, dopaminergic axons may integrate GABA_A_- and nAChR-mediated signals to independently make a computation that reflects striatal network activity and dopaminergic neuronal excitability (Brill-Weil et al., 2024).

GABA_B_ is also an important regulator of dopamine and has been shown in *ex vivo* brain slice experiments to suppress evoked release. Recent findings reveal that low threshold spiking interneurons gate dopamine release in a local and direct fashion through the activation of GABA_B_ receptors on dopaminergic axons (Holly et al., 2021). These results also suggest that this mechanism is independent of CIN input, allowing for precise, and direct, regulation of dopamine release.

#### Dopaminergic autoinhibition

Inhibitory D2Rs are expressed at dopaminergic terminals where they are canonically activated by dopamine signaling to inhibit further dopamine release (autoinhibition). Recent findings indicate D2Rs may also respond to presynaptic voltage, allowing them to directly sense bursts of action potentials (Sun et al., 2024). This autoregulatory signaling provides essential feedback control of dopamine levels in the striatum. D2R activation inhibits dopamine release through myriad mechanisms. D2Rs inhibit synthesis and vesicular packaging of dopamine (Onali et al., 1988), rapidly reduce excitation and Ca^2+^ influx (Stamford et al., 1991), potentiate K_v_1 channels (Congar et al., 2002), and activate G protein-coupled inwardly rectifying K^+^ (GIRK) channels to inhibit somatic activity (Ford et al., 2006). D2R-mediated GIRK currents are larger in somas of SNc than VTA dopaminergic neurons, suggesting a subregion-specific modulation of dopamine release by D2Rs. However, D_2_R-mediated inhibition of dopamine release from axon terminals is significantly more sensitive to dopamine, with little difference in efficacy between the DS and NAc (Cragg and Greenfield, 1997). This suggests that the axon is uniquely sensitive to autoregulatory inputs, which may reflect the axonal mechanisms mediating inhibition of release. There may also be a role for GIRK in inhibiting axonal excitability. In one study it was found that D_2_R-mediated inhibition of release was blunted by GIRK channel antagonists. The authors concluded that axonal K^+^ channels directly inhibit the secretory process, suggesting that Ca^2+^ channel modulation is not the sole mechanism by which release of neurotransmitter at the axon terminals is controlled (Congar et al., 2002).

The dopamine transporter (DAT) is a neurotransmitter transporter and Na^+^/Cl^−^ symporter. The main role of DAT is to transport dopamine into the presynaptic terminal, clearing it from the synapse. When DATs are inhibited, this leads to spillover and diffusion of dopamine transmission. The rate of dopamine uptake in the NAc is slower than the DS, producing subregion-specific dopamine-dependent signaling, with greater diffusion of dopamine in the NAc (Stamford et al., 1988). DATs also modulate axonal excitability through both electrogenic symporter currents and a small Cl^−^ conductance. The Cl^−^ conductance persists even in the presence of D1-, D2-, and α1-receptor antagonists, and reverses at a more depolarized potential than GABA_A_-mediated anion conductances in the same dopaminergic neurons (Ingram et al., 2002). Because this current was described in the cell body, it would be interesting to see if the same phenomena are observed in dopaminergic axons where this small Cl^−^ current could have large effects on axonal physiology. DATs may also play a role in the short-term plasticity of dopamine release through their electrogenic current. One study argues that DAT currents can modulate short-term depression via a K^+^-dependent gating mechanism. To specifically target DAT function, monoamine uptake inhibitors such as cocaine, methylphenidate, and nomifensine were observed to modulate short-term plasticity. The authors concluded that DATs limit the short-term depression of dopamine release in the striatal complex, operating as a “clamp” on dopamine transmission (Condon et al., 2019).

#### Hormone and neuropeptide modulation

The effects of sex hormones on the brain remain understudied, and there is a clear gap in the literature regarding the effects of sex hormones on axonal excitability. This biological variable is critical to investigate if we seek to understand how sex differences emerge across pathologies and psychological conditions. This information would help to further understand the cellular mechanisms by which sex differences emerge. Evidence of changing extracellular dopamine concentrations in conjunction with estrous cycle (Xiao and Becker, 1994) suggests that these hormones are important in the regulation of the basal ganglia. Indeed, many studies have shown that estradiol acts on estrogen receptors to modulate dopamine release from axon terminals, possibly through secondary cholinergic mechanisms (Almey et al., 2012; Almey et al., 2022; Yoest et al., 2019; Yoest et al., 2018). Progesterone is another ovarian hormone that fluctuates across an estrous or ovulatory cycle. Treatment of female rats with estradiol before progesterone administration enhances striatal dopamine release compared to estradiol alone (Becker and Rudick, 1999; Glaser et al., 1983; Cummings and Becker, 2012). Progesterone also promotes axonal myelination and repair (Schumacher et al., 2012). This could contribute to a neuroprotective effect in female individuals.

Dopamine also plays a critical role in regulating feeding behaviors. Neuropeptide Y (NPY) is a peptide neuromodulator with a broad range of physiological effects in both the central and peripheral nervous systems (Thorsell and Mathé, 2017; Li et al., 2019). NPY has been identified as particularly important in modulating feeding behaviors through control of dopamine release in the NAc, exerting an orexigenic effect (Raghanti et al., 2023). Humans, compared to other primates, have greater densities of dopaminergic axons and NPY-containing axons in the NAc (Raghanti et al., 2023; Hirter et al., 2021). However, what effect NPY may have on axonal excitability remains unresolved.

### Exogenous modulators and drugs

A universal property of addictive substances is that they “hijack” normal learning via modulation of the dopaminergic system in the striatal complex (Sulzer, 2011). Although addictive substances differ structurally and pharmacologically, they uniformly uncouple phasic dopamine signaling from environmental cues in favor of drug-associated triggers. These drugs are associated with elevated dopamine in the VTA, and cause extended synaptic modulation in this region (Bellone and Lüscher, 2006; Faleiro et al., 2004; Ungless et al., 2001). As with other modulatory substances, most studies have focused on somatic effects or release of transmitters. In this section, we will review common addictive substances and pathways by which they may affect axonal action potential transmission and dopamine release.

#### Psychostimulants

Cocaine is an addictive drug that acts as a stimulant, increasing locomotion in a dopamine-dependent manner (Wang et al., 2023). This drug acts on the dopaminergic system by blocking DAT, leading to increased and prolonged dopamine in the extracellular space that can potentiate D2R signaling (Rice and Cragg, 2008). Recently, DAT has been visualized in a human protein structure model interacting with cocaine (Nielsen et al., 2024). By blocking DAT, cocaine also modulates axonal excitability to lessen the short-term depression of dopamine release (Condon et al., 2019). Cocaine exposure can also profoundly alter the morphology of dopaminergic axons, leading to large-scale axonal re-arrangement after exposure to the drug. Cocaine exposure results in large, bulbous portions of the axon that are filled with mitochondria. Additionally, the authors observed increased axonal branching and pruning of bulbs on the axon reducing local connections (Wildenberg et al., 2021). Cocaine also resulted in swelling of the axonal bulb structures, similar to the structural changes seen in traumatic brain injuries (Wildenberg et al., 2021; Johnson et al., 2013).

Attention-deficit/hyperactivity disorder (ADHD) is a psychological disorder with a disease mechanism partially dependent on a hypoactive dopaminergic system. Currently, ADHD is treated with stimulants (like amphetamines or methylphenidate), which pose a risk for addiction or misuse. This is of concern especially in light of the genetic predisposition to addictive, impulsive, and compulsive behaviors of those with ADHD (Blum et al., 2008). Methamphetamines act as a substrate for monoamine transporters, like DAT, and increase dopamine release, while methylphenidate inhibits DAT. These effects on DAT produce modulation of axonal function due to altered electrogenic currents, which may contribute to the effects of these drugs on dopamine release. Repeated methamphetamine administration to mice causes significant degradation of SNc neurons and their striatal axons (Ares-Santos et al., 2014). Interestingly, amphetamine use also disrupts axonal growth, with sex-specific effects only in early adolescent male mice. Meanwhile, female mice experienced compensatory changes via Netrin-1 that helped to protect against axonal damage (Reynolds et al., 2023).

#### Nicotine

Cigarette smoking remains a leading cause of preventable disease in the United States and other countries (Benowitz, 2010). Beyond cigarettes, nicotine is now sold to consumers as oral nicotine products (like gums and lozenges) and e-cigarettes/vapes (Harlow et al., 2025). Emerging evidence suggests these new methods of nicotine consumption have long-term effects on respiratory, cardiovascular, and oral health (Meister et al., 2025). Still, there remain unanswered questions about how nicotine alters brain function to produce addiction. Highly debated is whether nAChRs on dopaminergic axons become desensitized to nicotine exposure, contributing to the development of habitual nicotine use. In the cell body, application of nicotine at a concentration relevant to human smokers only partially desensitized nAChR signaling, producing increased action potential firing (Pidoplichko et al., 1997). Since the exogenous application of nicotine to axon terminals alters CIN modulation of dopamine release, it is important to consider how these perturbations affect dopamine transmission. Foundational work showed that nicotine facilitates burst firing-mediated dopamine release at axon terminals in the striatum, suggesting that endogenous nAChR signaling in dopaminergic axons limits the transmission of action potential bursts (Rice and Cragg, 2004; Zhang and Sulzer, 2004). However, the cellular mechanism underlying this effect remains unresolved.

The β2 subunit of nAChRs is highly expressed on dopaminergic axons and is essential for the binding of nicotine. Knockout of the β2 subunit in dopaminergic neurons abolishes the nicotine-mediated release of dopamine *in vivo* and reduces nicotine self-administration (Picciotto et al., 1998). It has also been suggested that chronic nicotine may decrease the function of α6 containing nAChRs selectively in the NAc (Exley et al., 2013). Finally, investigating both the efficacy and recovery time of nicotine binding to axonal nAChRs is essential for understanding how nicotine use leads to substance use disorder. High concentrations of nicotine cause rapid dopamine release, which quickly diminishes within one minute. Subsequent nicotine exposures result in little additional dopamine release, indicating that nAChR desensitization leads to a form of cellular “memory” of prior exposure (Rowell and Duggan, 1998).

### Parkinson’s disease

A large component of the susceptibility of dopaminergic neurons to degenerate in Parkinson’s disease stems from the morphology and physiology of their axons. It is often observed that the axon is the first cellular compartment to degrade in SNc dopaminergic neurons in Parkinson’s patients (Cheng et al., 2010). In this section, we will discuss the relationship between axonal excitability and Parkinson’s disease pathology.

Parkinson’s disease (PD) is a neurodegenerative disease characterized by cell death in certain neuronal populations, including dopaminergic neurons. These neurons are unmyelinated or lightly myelinated. This renders them more susceptible to cellular stress due to their increased energy demands and distributed ion channel localization (Rasband et al., 1999). This is because one function of myelin is to decrease the energy demand required to transmit action potentials (Hildebrand et al., 1993). Substantial evidence indicates that dopaminergic axons are one of the first cellular structures to degrade (Kurowska et al., 2016). This axonal degradation leads to loss of striatal dopamine concentration and subsequent motor and cognitive symptoms. The reasons for dopamine neuron degradation are incompletely understood but often include metabolic factors that maintain axonal function (Bolam and Pissadaki, 2012). One axonal function is the maintenance of ion gradients, including those ions transmitted through nAChRs. One study found that the global expression of α3*/α6* nAChRs in the striatum is preferentially reduced following acute nigrostriatal degeneration. However, nicotine-evoked dopamine release remains around control levels in the DS, even though DAT protein levels, and thus dopamine uptake, are significantly reduced. These data suggest that the nAChRs on dopaminergic axons may compensate for the loss of dopaminergic fibers to boost dopamine release and maintain striatal function (McCallum et al., 2006). A foundational hypothesis states that acetylcholine and dopamine act in balance and competition in the DS, with evidence as far back as the 19th century when Jean Martin Charcot treated PD patients with an anticholinergic (Aosaki et al., 2010). Therefore, a clear link exists between acetylcholine and dopamine signaling in the striatum that is particularly relevant to the progression and treatment of Parkinson’s disease. This link warrants further investigation into how cholinergic modulation of axonal excitability and physiology is altered in Parkinson’s patients.

## Conclusion

Here we have discussed the many ways that dopaminergic axonal excitability is finely controlled and locally modulated. This control of dopaminergic axons is one of many ways that is used to control the amplitude and timing of dopamine release. As we move into an era of precision medicine, it becomes increasingly important to recognize the modulatory potential of the axon as a target for pharmaceutical intervention. Advancing interventions for diseases of the dopaminergic system demands incorporation of axonal control over dopamine release, underpinned by a comprehensive understanding of the intrinsic modulatory mechanisms at play.
